# NF-κB potentiates tumor growth by suppressing a novel target LPTS

**DOI:** 10.1186/s12964-017-0196-8

**Published:** 2017-10-10

**Authors:** Dongbo Liu, Hongping Miao, Yuanyin Zhao, Xia Kang, Shenglan Shang, Wei Xiang, Rongchen Shi, Along Hou, Rui Wang, Kun Zhao, Yingzhe Liu, Yue Ma, Huan Luo, Hongming Miao, Fengtian He

**Affiliations:** 1 Department of Biochemistry and Molecular Biology, Third Military Medical University, Chongqing, 400038 China; 2 Department of Neurosurgery, Southwest Hospital, Third Military Medical University, Chongqing, 400038 China

**Keywords:** NF-κB, LPTS, Promoter, Cervical cancer, Colon cancer

## Abstract

**Background:**

Chronic inflammation is causally linked to the carcinogenesis and progression of most solid tumors. LPTS is a well-identified tumor suppressor by inhibiting telomerase activity and cancer cell growth. However, whether and how LPTS is regulated by inflammation signaling is still incompletely elucidated.

**Methods:**

Real-time PCR and western blotting were used to determine the expression of p65 and LPTS. Reporter gene assay, electrophoretic mobility shift assay and chromatin immunoprecipitation were performed to decipher the regulatory mechanism between p65 and LPTS. Cell counting kit-8 assays and xenograt models were used to detect p65-LPTS-regulated cancer cell growth in vitro and in vivo, respectively.

**Results:**

Here we for the first time demonstrated that NF-κB could inhibit LPTS expression in the mRNA and protein levels in multiple cancer cells (e.g. cervical cancer and colon cancer cells). Mechanistically, NF-κB p65 could bind to two consensus response elements locating at −1143/−1136 and −888/−881 in the promoter region of human *LPTS* gene according to EMSA and ChIP assays. Mutation of those two binding sites rescued p65-suppressed *LPTS* promoter activity. Functionally, NF-κB regulated LPTS-dependent cell growth of cervical and colon cancers in vitro and in xenograft models. In translation studies, we verified that increased p65 expression was associated with decreased LPTS level in multiple solid cancers.

**Conclusions:**

Taken together, we revealed that NF-κB p65 potentiated tumor growth via suppressing a novel target LPTS. Modulation of NF-κB-LPTS axis represented a potential strategy for treatment of those inflammation-associated malignancies.

## Background

Chronic inflammation is causally linked to the carcinogenesis and development of most solid tumors [1–5]. The proinflammatory signal in cancers is characterized by the activated inflammatory pathways (e.g. NF-κB and JNK), elevated inflammatory cytokines (e.g. IL-1β, IL-6 and TNFα) and increased infiltration of immune cells (e.g. macrophages and lymphocytes) [6–8]. In fact, the inflammatory microenvironment caused by microbial infection plays a very important role in malignant transformation and cancer progression. For example, cervix infection by HPV16 is an independent and high risk factor of cervical cancer [9]. Helicobacter pylori-mediated chronic atrophic gastritis is suggested to be a precancerosis of gastric cancer [10]. Similarly, imbalance of intestinal microbiota is also causally linked to intestine inflammation and tumors [11, 12].

It has been revealed that NF-κB is a potent contributor in cancer progression by enhancing the expression of oncogenes and activation of onco-signals [13]. As a transcription factor, NF-κB controls the transcription specificity via the assembly of heterodimers or homodimers of five different NF-κB proteins (p65, p50, c-Rel, p105 and p100) [14]. In response to proinflammatory stimulation, the I-κB kinase is activated to phosphorylate I-κB protein and suppress I-κB-mediated p65 degradation [15]. P65 contains a DNA binding domain and mediates the transcription function of NF-κB in most situations [16].

LPTS, also named PINX1, is a well-characterized tumor suppressor by inhibiting telomerase activity in multiple cancers [17, 18]. It has been reported that LPTS expression is deficient in multiple cancers and positively correlated to prognosis [19–21]. Mechanistically, LPTS inhibited tumor cell growth by directly suppressing the activity of human telomerase reverse transcriptase (hTERT) or reducing c-myc-mediated hTERT transcription [22, 23]. However, how the expression of LPTS is repressed in a special cancer type is still incompletely elucidated.

Modulation of inflammatory signaling is associated with alteration of some important regulators in cancer progression (e.g. c-myc and p53) [24]. We previously reported that HPV16 E6 could suppress p53-dependent LPTS expression in cervical cancer cells [25]. Provided that HPV16 E6 is also a proinflammatory stimulator [26], we aimed to investigate whether inflammatory signals would regulate LPTS expression in inflammation-associated cancer cells (e.g. cervical cancer and colon cancer). We would also investigate the regulatory mechanism of LPTS in vitro and in xenograft models.

## Methods

### Cell culture

CaSki cell, a HPV16/HPV18-positive cervical carcinoma cell line, and MKN-45 cell, a human gastric carcinoma cell line, were purchased from American Type Culture Collection (Rockville, MD, USA). MC-38, a mouse colorectal cancer cell line, was maintained in our lab [27]. All the cells had been authenticated and tested for mycoplasma. Those cells were grown in high glucose DMEM supplemented with 10% fetal bovine serum (FBS), penicillin G (100 units/ml) and streptomycin (100 μg/ml) at 37 C° in a humidified 5% CO_2_ atmosphere. Cells at approximately 80% confluence were washed with PBS and preincubated in serum-free medium for 2 h before treatment with the NF-κB inhibitor BAY11–7082 (S1523, Beyotime, Shanghai, China).

### Mice

All the mouse experiments were approved by the Institutional Animal Care and Use Committee of the Third Military Medical University and carried out in accordance with the "Guide for the care and use of laboratory animals" published by the US National Institutes of Health (Publication no.85–23, revised 1996). All the mice were purchased from the Institute of Experimental Animal in Third Military Medical University (Chongqing, China). Each group of mice was maintained five per cage supplied with a regular rodent diet and standard water ad libitum in a pathogen-free facility with a 12-h light, 12-h dark cycle.

### Subcutaneous xenograft models

Eight weeks old female BALB/c nude mice were subcutaneously inoculated with CaSki cells or MKN-45 cells (5.0 × 10^6^/100 μl PBS) in the groin. 6–8 weeks old female C57BL/6 mice were subcutaneously injected with MC-38 cells (5.0 × 10^6^/100 μl PBS) in the groin. All the inoculated cancer cells were stably transfected with shRNAs of LPTS or p65. For overexpression of E6, the CaSki cells were transfected transiently. The NF-κB inhibitor BAY11–7082 was injected intraperitoneally at 5 mg/kg dissolved in DMSO/PBS buffer thrice per week. The control group received the equal volume of vehicle only. The volume of the tumor was determined dynamically as described previously [27]. The survival time of those mice were also recorded.

### Transfection

Transfection was performed according to the protocol of Lipofectamin-2000 (#11668019, Invitrogen, Shanghai, China). Briefly, cells were plated 24 h before transfection at a density of 1.0 × 10^5^/well on a 24-well plate (#CLS3524, Sigma, Shanghai, China). CaSki or MC-38 cells were incubated 4 h before transfection with FBS-free, antibiotic-free media and then transfected with plasmids (0.2 μg/ml) or shRNAs (20 nmol/ml). After transfection for 6 h, the medium was removed and replaced with complete growth medium for further treatment. For overexpression of HPV16-E6 in the CaSki cells, the plasmid pEGFP-HPV16-E6 or the control vector pEGFP-N1 was constructed and transiently transfected respectively, as described in our previous report [25].

### Packaging and transfection of lentiviruses

P65 and LPTS shRNA constructs for lentivirus packaging were purchased from Sigma. The sequences used for human p65 silence were: 5′- CCGGAGAGGACATTGAGGTGTATTTCTCGAGAAATACACCTCAATGTCCTCTTTTTTG-3′ (p65-shRNA-1); 5′- CCGGCCCTGAGCACCATCAACTATGCTCGAGCATAGTTGATGGTGCTCAGGGTTTTTG-3′ (p65-shRNA-2). The sequences used for human LPTS silence were: 5′- CCGGGAGACGCAGGTGGAACGTAAACTCGAGTTTACGTTCCACCTGCGTCTCTTTTTG -3′ (hLPTS-KD). The sequences used for mouse p65 silence were: 5′- CCGGCCCTCAGCACCATCAACTTTGCTCGAGCAAAGTTGATGGTGCTGAGGGTTTTTG -3′ (mp65-KD). The sequences used for mouse LPTS silence were: 5′- CCGGCCGGGTTCATTATATGAAATTCTCGAGAATTTCATATAATGAACCCGGTTTTTG-3′ (mLPTS-KD). The control shRNA plasmid was also provided by Sigma (#SHC016-1EA). The Caski cells and MC-38 cells with stable silence of p65, LPTS or both were constructed for the assays of cell growth in vitro and in vivo.

### Reporter gene constructs and assays

The reporter gene pGL3-C1harboring the promoter region (−2303/+42) of human *CCL20* was used to indicate the transcription activity of NF-κB, as a NF-κB binding element (GGGGAAAACCCC) locating at −81/−70 was identified in our previous study [28]. The pGL3-basic vector-based reporter gene containing the human LPTS promoter region −1300/+25 or −495/+25 was constructed. Two potential NF-κB binding sites locating at −1143/−1136 (tgggaaaa) and −888/−881 (tggagagt) in the human LPTS promoter region were mutated to MUT-1(tgtctaaa) and MUT-2 (tgcatagt) respectively or simultaneously (MUT-3) in the reporter constructs according to a site-directed mutation protocol (#D401, TaKaRa MutanBEST Kit, TaKara, Japan) as described previously [28] (See in Fig. 3c). The mutation primers were designed as follows: for MUT-1, the forward: 5′-ttggctgtctaaattccattcact-3′, the reverse: 5′-ggcaggaaagctgtgacattgtga-3′; for MUT-2, the forward: 5′-ttactgcatagtcactcacccaa-3′, the reverse: 5′-acttcaggtgacagtgcacaca-3′. Each reporter construct was transfected into the CaSki cells for more than 24 h. The cells were washed twice with PBS and lysed with specific reporter lysis buffer. Then, the luciferase activities of the cell lysate were evaluated according to the manufacturer’s instructions (#E1910, Promega, Shanghai, China), and the total protein concentration in each well was measured as an internal control. The final results were displayed as relative activities.

### Reverse transcription-quantitative polymerase chain reaction (RT-qPCR)

Total RNA was isolated using TRIzol reagent (Invitrogen; Thermo Fisher Scientific, Inc.). 1 μg RNA was reverse transcribed into cDNA using the RevertAid First Strand cDNA Synthesis kit (#K1622, Thermo Fisher Scientific, Inc.) according to the manufacturer’s protocol. qPCR was carried out using a ABI 7500 Real-Time PCR system (Applied Biosystems; Thermo Fisher Scientific, Inc.). The mRNA expression levels were normalized to β-actin. Reactions were performed in duplicate using a SYBR kit (TakaRa, Shiga, Japan) The primers were designed and synthesized upon request. The amplification steps consisted of denaturation at 95 °C, followed by 40 cycles of denaturation at 95 °C for 15 s and then annealing at 60 °C for 1 min. Relative target gene expression was calculated using the 2^-ΔΔCq^ method [29].

### Western blotting

Cells were lysed with RIPA Lysis and Extraction Buffer (#P0013C, Beyotime, Shanghai, China) supplemented with 1% protease inhibitor cocktail (#P8340, Sigma), as well as 1 mM phosphatase inhibitors (#2850, Sigma) and shaken for 30 min before centrifugation at 12000 g for 30 min at 4 °C. The supernatant was collected and quantified using a BCA kit (#P0009, Beyotime, Shanghai, China). The extracted proteins (50 μg/well) were separated through SDS-PAGE on a 10% gel, and transferred to a polyvinylidene difluoride membrane. The membrane was blocked with 5% non-fat milk at 4 °C overnight, and then incubated with anti-LPTS (#H00054984-K, Abnova), anti-p65 (#8242, Cell signaling) and anti-β-actin (#3700, Cell signaling) for 10 h at 4 °C. The membrane was rinsed 3 times with PBS containing 0.1% Tween 20 (PBST), and incubated with the appropriate horseradish peroxidase-conjugated second antibody for 1 h at room temperature. The membrane was then washed with PBST for 3 times and incubated with enhanced chemiluminescence substrate (#NEL105001EA, PerkinElmer) for 1 min at room temperature. The signals were captured using a ChemiDoc Touch™ Imaging system (Bio-Rad Laboratories).

### Electrophoretic mobility shift assay (EMSA)

The nuclear extracts were prepared from the empty vector pEGFP-N1 or pEGFP-HPV16-E6 transfected -CaSki cells. EMSA was performed according to the manufacturer’s instruction (#GS002, Beyotime, Shanghai, China). The biotin-labeled probes harboring the potential NF-κB binding elements (−1150/−1125, 5′-CCTTGGCTGGGAAAATTCCATTCACT-3′; −895/−870, 5′- AGTTTACTGGAGAGTCACTCACCCAA -3′) were designed and synthesized (Sbsgene, Shanghai, China). The underlined bases indicated the core sequences.

### Chromatin immunoprecipitation (ChIP)

The binding activity between p65 protein and the potential NF-κB binding elements (−1143/−1136, 5′-TGGGAAAA-3′; −888/−881, 5′-TGGAGAGT-3′) in the promoter region of human LPTS in CaSki cells was measured using the ChIP test. Briefly, CaSki cells were fixed with 1% of formaldehyde and then lysed in cell lysis buffer (5 mM PIPES, 85 mM KCl, and 0.5% NP-40, supplemented with protease inhibitors, pH 8.0) using a dounce homogenizer to isolate the nuclei. The nuclei were resuspended in nuclei lysis buffer (50 mM Tris-HCl, 20 mM EDTA, and 1% SDS, supplemented with protease inhibitors, pH 8.1) and sonicated to shear genomic DNA to an average fragment length of 200–1000 bp. Lysates were centrifuged, and the supernatants were collected. 50 μl of each samples was used as the input control. The supernatants underwent overnight immunoprecipitation (with IgG or p65), elution, reverse cross-lining and protease K digestion according to manufacturer’s protocol (#P2078, Beyotime, Shanghai, China). Purified DNA extracts were analyzed by realtime PCR using the primer pairs that cover the predicted NF-κB binding elements in the LPTS promoter region. The primers for element (−1143/−1136) were 5′-CCTTCCTGAGTCCAGTGC-3′ (forward) and 5′-GGGAGGCAAGTGAATGGAA-3′ (reverse). The primers for element (−888/−881) were 5′-CTGAAGTTTACTGGAGAG-3′ (forward) and 5′- CAGGGGAGTTCTAATAAG-3′ (reverse). The primers for input control were 5′-TTCACTTGCCTCCCCTCAC-3′ (forward) and 5′-ACTCAGGTGCCAAGAAAAGC-3′ (reverse).

### Cell counting kit-8 (CCK8) assays

The cell viability of CaSki and MC-38 cells were measured by CCK8 assays. The cells were seeded at 1.0 × 10^4^ cells/well in 96-well plates. After overnight incubation at 37 C° in a humidified 5% CO_2_ atmosphere, the medium was removed and replaced with100 μl of PBS containing 20 μl of CCK8 solution (5 g/l, Sigma, USA). Plates were then incubated at 37 C° for 3 h before measurement (Time point 0) using a microculture plate reader at a wavelength of 450 nm. Then, the testing solution was removed and the cells were subjected to further treatment of NF-κB inhibitor (10 μM) or DMSO as control. CCK8 assays were carried out at time point 24 and 48 h. The cell growth curve was obtained according to the readout at the wavelength of 450 nm at different time points.

### Statistical analysis

Statistical analysis was performed using GraphPad Prism version 5.01 (GraphPad Software, Inc., La Jolla, CA, USA). The results were expressed as the mean ± standard error of the mean and were analyzed using a two-tailed unpaired Student’s t-test or one-way ANOVA analysis of variance followed by post hoc test for multiple comparisons. *P* < 0.05 indicated a statistically significant difference.

## Results

### Expression of LPTS is generally reduced in cancer tissues

**Fig. 1:**
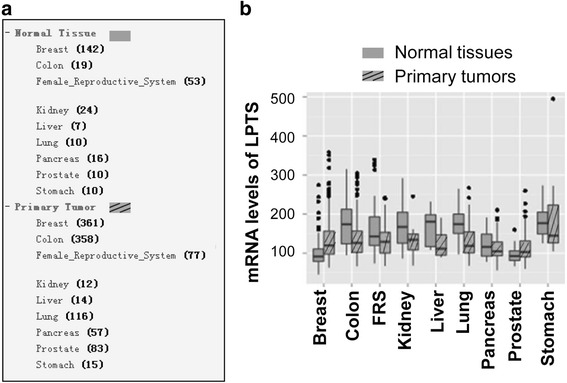
mRNA expression of LPTS is generally reduced in cancer tissues. **a** The sample size of the normal tissues and primary tumors in selected human organs in an open database (http://merav.wi.mit.edu/SearchByGenes.html). **b** mRNA expression of LPTS in cancer and normal tissues. FRS, female reproductive system

It’s reported that LPTS is a tumor suppressor in multiple cancers [19–21, 23]. From an open database (http://merav.wi.mit.edu/SearchByGenes.html), we demonstrated that the mRNA level of LPTS was generally decreased in cancer tissues of colon, female reproductive system, kidney, liver, lung, pancreas and stomach, although the LPTS expression is increased in breast and prostate cancers (Fig. 1a -b). It’ reported that most of those cancers underwent an inflammatory process before malignant transformation [30–33]. This clue indicated a potential correlation between inflammatory signaling pathways and LPTS expression.

### NF-κB suppresses LPTS expression in mRNA and protein levels

**Fig. 2:**
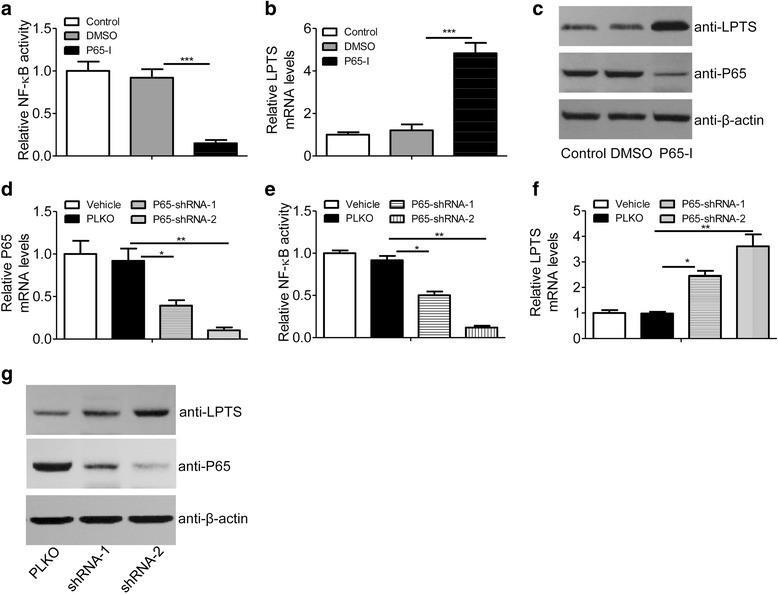
NF-κB suppresses LPTS expression in mRNA and protein levels. **a** Relative NF-κB activity in CaSki cells. CaSki cells were transfected with a reporter gene containing consensus binding elements of NF-κB and treated with a NF-κB inhibitor BAY11–7082 (10 μM) for 24 h. Cells were harvested for luciferase activity assays. (*n* = 4, ***P < 0.005). **b** Relative mRNA levels of *LPTS* in CaSki cells treated with BAY11–7082 (10 μM) for 24 h. (*n* = 4, ***P < 0.005). **c** Immunoblotting assays of LPTS and P65 in CaSki cells as described in (**b**). Representative results were displayed. **d** Relative mRNA expression of *LPTS* in CaSki cells transfected with p65 shRNAs (shRNA-1 or shRNA-2) or PLKO vector as control. (*n* = 4, *P < 0.05 and **P < 0.01). **e** Relative NF-κB activity in CaSki cells transfected with p65 shRNAs or PLKO vector as control. (*n* = 4, *P < 0.05 and **P < 0.01). **f** Relative mRNA levels of *LPTS* in CaSki cells transfected with p65 shRNAs or PLKO vector as control. (*n* = 4, *P < 0.05 and **P < 0.01). **g** Immunoblotting assays of LPTS and P65 proteins in CaSki cells transfected with p65 shRNAs or PLKO vector as control. Representative results were displayed. All the histograms in this figure show the means ± s.e.ms., Student’s *t*-test

The classic inflammatory pathways include NF-κB and JNK [34]. To investigate whether inflammatory signaling would regulate LTPS expression, a HPV16/HPV18-positive cervical carcinoma cell line (CaSki cell) was employed, as NF-κB was activated in response to virus infection [35]. We demonstrated that the NF-κB inhibitor markedly suppressed NF-κB activity (Fig. 2a) and potentiated LPTS expression in mRNA and protein levels in CaSki cells (Fig. 2b-c). However, the JNK inhibitor didn’t affect the expression of LPTS significantly (Data not shown). For confirmation, we further silenced p65 expression and reduced NF-κB activity with p65-specific shRNAs (Fig. 2d-e). Obviously, p65 shRNA-2 was more efficient than shRNA-1. As expected, p65 shRNAs markedly increased the expression of LPTS in mRNA and protein levels (Fig. 2f-g).

### NF-κB suppresses LPTS expression through cis-regulatory elements

**Fig. 3:**
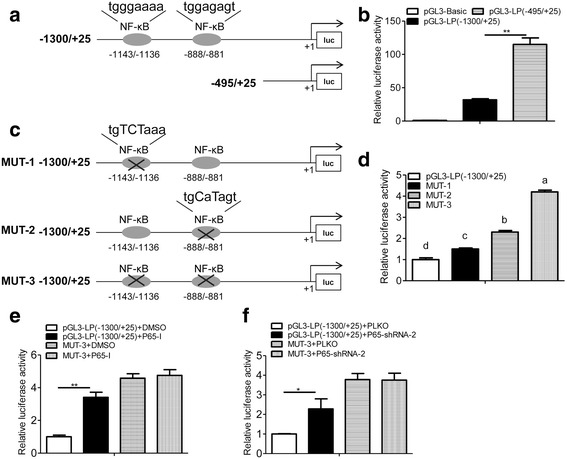
NF-κB suppresses LPTS expression in the promoter level. **a** Schematic diagram of two constructs harboring the promoter regions (−1300/+25; −495/+25) of human *LPTS* gene. Two putative NF-κB binding elements locating at −1143/−1136 (tgggaaaa) and −888/−881 (tggagagt) were displayed. **b** Relative luciferase activity of CaSki cells transfected with pGL3-Basic, pGL3-LP(−495/+25) or pGL3-LP(−1300/+25) for 24 h.(*n* = 4, **P < 0.01, means ± s.e.ms., Student’s *t*-test.). **c** Schematic diagram of reporter constructs harboring the human *LPTS* promoter (−1300/+25) with different mutant elements for NF-κB as indicated. **d** Relative luciferase activity of CaSki cells transfected with pGL3-LP(−1300/+25), MUT-1, MUT-2 and MUT-3, respectively (*n* = 4, means ± s.e.ms., one-way ANOVA). Values not sharing a common superscript letter differ significantly. **e** The NF-κB binding elements were responsible for the activity of LPTS promoter. CaSki cells were transfected with pGL3-LP(−1300/+25) or MUT3, and then treated with BAY11–7082 (10 μM) for 24 h. Cells were harvested for luciferase activity assays. (*n* = 3, **P < 0.01, means ± s.e.ms., Student’s *t*-test.). **f** Relative luciferase activity of CaSki cells transfected with pGL3-LP(−1300/+25) or MUT3 plus with PLKO or P65-shRNA for 24 h. (*n* = 3, *P < 0.05, means ± s.e.ms., Student’s *t*-test)

Provided that NF-κB is a transcription factor and that two putative NF-κB binding elements locating at −1143/−1136 and −888/−881 in the promoter region of human *LPTS* gene were predicted according to an online-software (http://alggen.lsi.upc.es/), we next deciphered whether NF-κB would regulate LPTS expression in the promoter level. We constructed two reporter plasmids: one harboring the promoter region −1300/+25 containing both potential NF-κB binding sites, the other harboring the region −495/+25 without any predicted NF-κB binding elements (Fig. 3a). Those reporter plasmids were transfected into CaSki cells and luciferase activity was determined. We demonstrated that the reporter gene activity of −495/+25 region was notably higher than that of the −1300/+25 region (Fig. 3b), indicating that those two predicted binding sites of NF-κB might be functional in regulating LPTS promoter activity. Thus, we next performed site-directed mutation of those two binding sites respectively or simultaneously, and further evaluated their effects on LPTS promoter activity (Fig. 3c). We revealed that simultaneous mutation of those two sites greatly increased the reporter gene activity and the proximal site (−888/−881) was more profound than the distal one (−1143/−1136) (Fig. 3d). Especially, inactivation of NF-κB with a specific inhibitor or p65 shRNA markedly stimulated promoter activity of LPTS, while simultaneous mutation of both NF-κB binding elements fully abrogated this effect (Fig. 3e-f).

### NF-κB (p65) directly binds to *LPTS* promoter

**Fig. 4:**
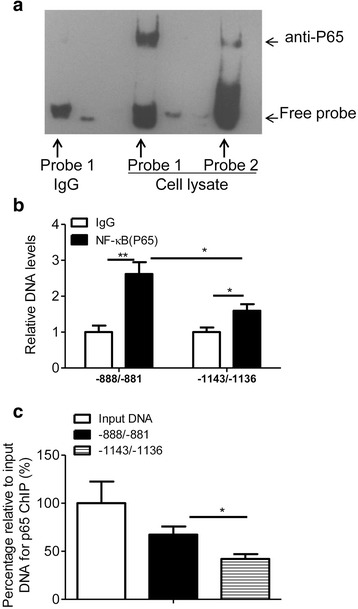
NF-κB directly binds to *LPTS* promoter. **a** EMSA was conducted to examine the DNA binding activity of NF-κB. Probe 1 contains the potential NF-κB binding site locating at −888/−881, and probe 2 contains another NF-κB binding site at −1143/−1136 in the human *LPTS* promoter. Representative images were shown. **b** ChIP assays were performed to determine the binding activity between NF-κB and the potential elements (−888/−881; −1143/−1136) in the human *LPTS* promoter in CaSki cells (*n* = 4, *P < 0.05, **P < 0.01, means ± s.e.ms., Student’s *t*-test). **c** Percentage relative to input DNA for p65 ChIP. CaSki cells were treated and harvested as described in (**b**). (n = 3, *P < 0.05, means ± s.e.ms., Student’s *t*-test)

To verify the direct binding between NF-κB (p65) and its potential binding sites in LPTS promoter, EMSA and ChIP assays were performed. In vitro, we demonstrated that probe 1 harboring the proximal site (−888/−881) incorporated more p65 proteins than probe 2 containing the distal site (−1143/−1136) (Fig. 4a). In vivo, the binding activity between p65 proteins and the predicted binding elements were confirmed by ChIP assays. Consistent with the EMSA test, we revealed that the binding activity of p65 proteins to the proximal site was more profound than the distal one (Fig. 4b-c).

### HPV16-E6 promotes NF-κB-dependent growth of CaSki cells

**Fig. 5:**
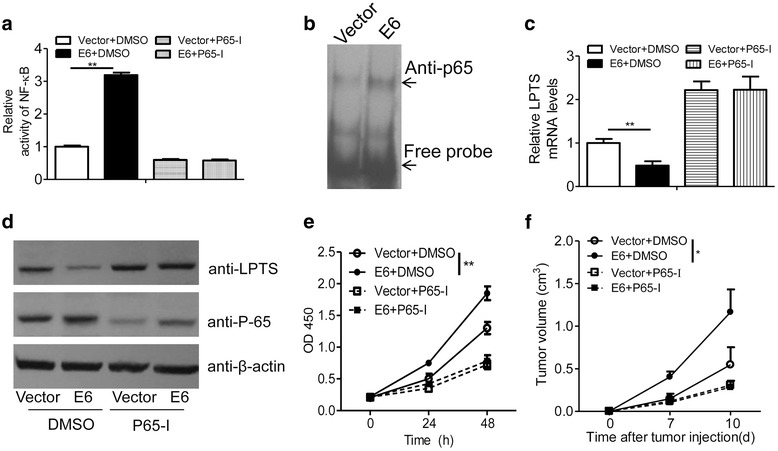
HPV16-E6 promotes NF-κB-dependent growth of CaSki cells. **a** HPV16-E6 stimulates the activity of NF-κB. The empty vector or pEGFP-HPV16-E6-transfected CaSki cells were additionally transfected with a reporter gene which contains consensus binding elements of NF-κB, and then treated with a NF-κB inhibitor BAY11–7082 (10 μM) for 24 h. Cells were then harvested for luciferase activity assays (*n* = 4, **P < 0.01, means ± s.e.ms., Student’s *t*-test). **b** EMSA was conducted to examine the binding activity between p65 and probe 1 which contains a potential NF-κB binding site locating at −888/−881 in the promoter of human *LPTS.* The cell lysates were collected from the empty vector or HPV16-E6-transfected CaSki cells. Representative results were displayed. **c** Relative mRNA expression of LPTS in the empty vector or HPV16-E6-transfected CaSki cells which were treated with DMSO or a NF-κB inhibitor BAY11–7082 (10 μM) for 24 h. (*n* = 4, **P < 0.01, means ± s.e.ms., Student’s *t*-test). **d** Immunoblotting assays of LPTS and p-65 proteins in the cells as described in (**c**). **e** CCK8 assays of the CaSki cells. The empty vector or HPV16-E6-transfected CaSki cells were treated with DMSO or a NF-κB inhibitor BAY11–7082 (10 μM). CCK8 assays were performed dynamically as indicated (*n* = 5, **P < 0.01, means ± s.e.ms., Student’s *t*-test). **f** Volume of the inoculated CaSki tumors. The empty vector or HPV16-E6-transfected CaSki cells were inoculated subcutaneously in the Balb/C nude mice and treated with DMSO or a NF-κB inhibitor BAY11–7082 every three days. The tumor size was measured dynamically (*n* = 5, *P < 0.05, means ± s.e.ms., Student’s *t*-test)

Aforementioned results revealed the inhibitory role of NF-κB in LPTS expression. However, the function of NF-κB-LPTS axis in tumor progression was still not elucidated. Human papillomavirus 16 (HPV16) was causally linked to the carcinogenesis and progression in most of the cervical cancers. It’s reported that the oncogene E6 from HPV16 could stimulate NF-κB activity in multiple mechanisms. We demonstrated that E6 transfection largely increased the transcription activity of NF-κB in CaSki cells (Fig. 5a). Consistently, E6 transfection also potentiated the binding activity between the p65 protein and LPTS promoter in EMSA tests (Fig. 5b). Subsequently, E6 transfection dramatically suppressed LPTS expression in the mRNA and protein levels in a p65 dependent manner (Fig. 5c-d). We next explored the functional relevance of E6-NF-κB axis by measuring cancer cell growth in vitro and in xenograft models. E6 transfection significantly induced CaSki cell growth, and this effect was abrogated by additional treatment of a NF-κB inhibitor (Fig. 5e). Further, in subcutaneous xenograft models, we confirmed that E6 transfection potentiated NF-κB dependent CaSki tumor growth (Fig. 5f).

### NF-κB promotes LPTS-dependent growth of CaSki cells

**Fig. 6:**
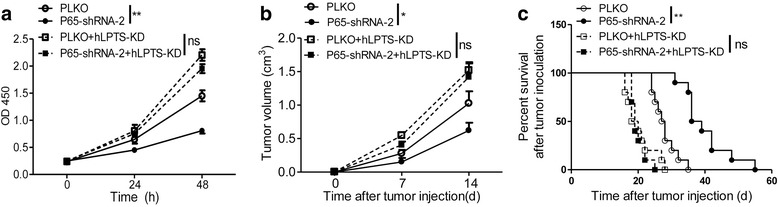
NF-κB promotes LPTS-dependent growth of CaSki cells. **a** CCK8 assays of the CaSki cells. The PLKO or P65 shRNA-2-transfected CaSki cells were additionally transfected with a human LPTS shRNA (hLPTS-KD). CCK8 assays were performed dynamically. (*n* = 5, **P < 0.01, means ± s.e.ms., Student’s *t*-test). **b** The size of the inoculated CaSki tumors. The CaSki cells were treated as described in (**a**), and injected subcutaneously in the Balb/C nude mice. The tumor size was measured dynamically as indicated. (*n* = 10, *P < 0.05, means ± s.e.ms., Student’s *t*-test). **c** Percent survival of the CaSki tumor-bearing mice. The Balb/C nude mice were inoculated with subcutaneous CaSki tumors as described in (**b**), and the survival time was recorded. (*n* = 10, **P < 0.01, means ± s.e.ms., Gehan-Breslow-Wilcoxon test)

To validate whether NF-κB regulates cervical cancer cell growth through repressing LPTS, the p65-silenced CaSki cells were additionally transfected with LPTS shRNAs. In CCK8 assays, repressed cell growth by p65 silence was largely rescued by LPTS knockdown (Fig. 6a). Consistently, silence of p65 significantly inhibited the growth of CaSki tumor, and this effect was prevented by additional knockdown of LPTS by shRNAs (Fig. 6b). We next investigated the survival time of the CaSki tumor-bearing mice, and demonstrated that p65 deficiency notably improved the survival of those tumor-bearing mice in a LPTS dependent manner (Fig. 6c).

### NF-κB promotes LPTS-dependent growth of colorectal cancer cells

**Fig. 7:**
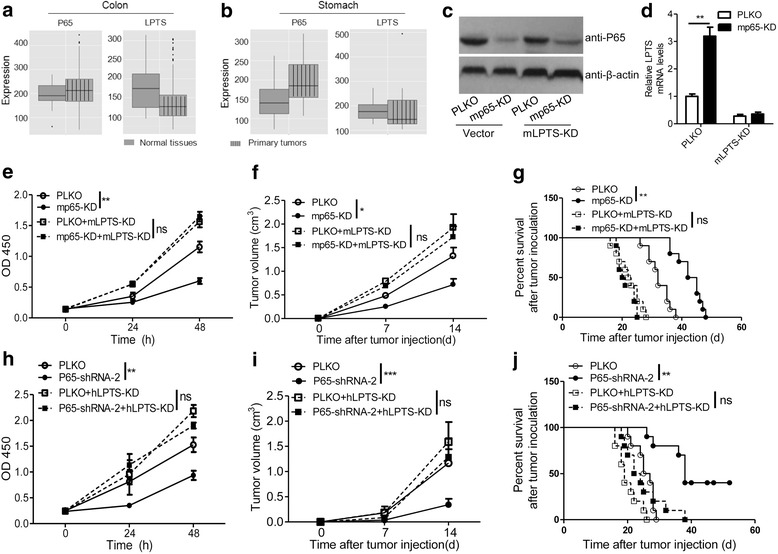
NF-κB promotes LPTS-dependent growth of cancer cells. **a** Relative mRNA Expression of human P65 and LPTS in the normal or primary tumor tissues in the colon. Data were obtained from an open database (http://merav.wi.mit.edu/SearchByGenes.html). The normal tissues include 38 samples and the tumor tissues include 716 samples. **b** Relative mRNA levels of human P65 and LPTS in the normal or primary tumor tissues in the stomach. Data were collected from the open database as described in (**a**). The normal tissues include 10 samples and the tumor tissues include 15 samples. **c** Immunoblotting assays of P65 in MC-38 cells. The PLKO or a mouse p65 shRNA (mP65-KD)-transfected MC-38 cells were additionally transfected with a vector control or a mouse LPTS shRNA (mLPTS-KD). The cells were harvested for western blotting assays. **d** mRNA expression of LPTS in MC-38 cells as described in (**c**) (*n* = 4, **P < 0.01, means ± s.e.ms., Student’s *t*-test). **e** CCK8 assays were performed on the MC-38 cells described in (**c**). (*n* = 5, **P < 0.01, means ± s.e.ms., Student’s *t*-test). ns, not significant. **f** Volume of the inoculated MC-38 tumors. The MC-38 cells described in (**c**) were subcutaneously inoculated in C57BL/6 mice and the tumor size was determined dynamically. (*n* = 5, *P < 0.05, means ± s.e.ms., Student’s *t*-test). ns, not significant. **g** Percent survival of the MC-38 tumor-bearing mice. The C57BL/6 mice were inoculated with subcutaneous MC-38 tumors as described in (**f**), and the survival time was recorded. (*n* = 10, **P < 0.01, means ± s.e.ms., Gehan-Breslow-Wilcoxon test). ns, not significant. **h** p65 silence inhibited gastric cancer cell growth in a LPTS dependent manner. The PLKO or P65-shRNA-2-transfected MKN-45 cells were additionally transfected with a vector control or a human LPTS shRNA (hLPTS-KD). The cell growth was measured by CCK8 assays. (*n* = 5, **P < 0.01, means ± s.e.ms., Student’s *t*-test). ns, not significant. **i** Volume of the inoculated MKN-45 tumors. The MKN-45 cells described in (**h**) were subcutaneously inoculated in rude mice and the tumor size was determined dynamically. (*n* = 5, ***P < 0.005, means ± s.e.ms., Student’s *t*-test). ns, not significant. **j** Percent survival of the MKN-45 tumor-bearing mice. The rude mice were inoculated with subcutaneous MKN-45 tumors as described in (**h**), and the survival time was recorded. (*n* = 10, **P < 0.01, means ± s.e.ms., Gehan-Breslow-Wilcoxon test). ns, not significant

Given the importance of NF-κB pathway in inflammation [36], we presumed that NF-κB-LPTS axis might play a dominant role not only in cervical cancer but also in other inflammation-associated tumors, such as gastric cancer and colon cancer. From the open database of gene expression in human cancer tissues (http://merav.wi.mit.edu/SearchByGenes.html), we found the expression of p65 was increased, while the level of LPTS was reduced in colon cancer tissues relative to the normal colons (Fig. 7a). A similar expression profile of p65 and LPTS as what we found in the colon cancer was also observed in gastric cancer tissues and normal ones (Fig. 7b). To further confirm the role of NF-κB-LPTS axis in cancer cell growth, we selected a mouse colorectal cancer cell line MC-38 and set up a subcutaneous xenograft model on C57BL/6 mice. In vitro, we verified that p65 silence potentiated LPTS expression in MC-38 cells (Fig. 7c-d). LPTS knockdown could fully rescue p65 silence-attenuated cell growth (Fig. 7e). In vivo, we demonstrated that p65 deficiency notably inhibited MC-38 tumor growth and improved the survival of those tumor-bearing mice in a LPTS dependent way (Fig. 7f-g). Furthermore, a human gastric cancer cell line MKN-45 was employed to confirm the function of NF-κB-LPTS axis in cancer cell growth. As expected, similar results as what we found in MC-38 cells were obtained in CCK8 assays and subcutaneous xenograft models (Fig. 7h-j).

## Discussion

Chronic inflammation and LPTS expression are associated with cancer development [12, 20]. However, the relationship between inflammation and LPTS expression is still not known. In the present study, we for the first time demonstrated that NF-κB (p65) inhibited LPTS expression by directly binding to two consensus response elements in the promoter region of LPTS. We also revealed that NF-κB (p65) potentiated LPTS-dependent cancer cell growth in vitro and in xenograt models. Clinical relevance study indicated that NF-κB-LPTS axis might be a potential target in cancer treatment.

NF-κB and JNK signaling are classic proinflammatory pathways in response to inflammatory stimuli [34, 37]. In the present study, we investigated those two pathways and identified NF-κB (p65) as a functional suppressor of LPTS expression. In fact, other inflammatory pathways (e.g. STAT signals [38] and inflammasomes [37]) still needed to be investigated in future studies. Perhaps, we could identify more inflammation response elements in the LPTS promoter.

Whether LPTS would regulate inflammation signals is another interesting question. According to our data shown in Fig. 7c, LPTS silence didn’t affect NF-κB p65 expression notably. Meanwhile, the transcription activity of NF-κB was also not regulated by LPTS (data not shown). Those findings indicated that LPTS didn’t regulate NF-κB signaling. However, whether and how the inflammatory cytokines (e.g. IL-1β, IL-6, TNFα and IL-10) or other inflammatory signals (e.g. JNK, Stat and inflammasome pathways) are influenced by LTPS still need to be investigated in future studies. High throughput studies like gene expression arrays or RNA sequencing would be helpful in answering the above questions.

LPTS, a potent inhibitor of telomerase activity, is a well-characterized tumor suppressor [17, 39]. LPTS inhibits proliferation, migration and invasion of cancer cells [40]. Reduced expression of LPTS correlates to the progressive features of cancers [21]. To the best of our knowledge, no drugs have been designed to modulate the expression of LPTS. Therefore, identifying the regulators of LPTS is essential for exploring novel targets for cancer therap. Here we identified that LPTS was negatively regulated by NF-κB, which could be inhibited by a series of specific inhibitors (e.g. BAY11–7082). Our findings indicated that the NF-κB inhibitors might be translated in cancer therapy. Indeed, emerging studies reported that modulation of LPTS expression could synergize the anti-tumor effects of regular chemotherapy drugs in clinic [41, 42].

It should be pointed out that not all the LPTS deficiency in cancers was resulted from inflammation stimuli. As shown in Fig. 1, LPTS expression was increased in breast and prostate cancer tissues. These findings revealed a complex regulatory mechanism of LPTS expression. As reported previously in cervical cancer cells, we demonstrated that LPTS was also a target gene of p53 [25], a transcription factor which was generally inactivated in multiple cancer cells [43, 44]. It’s very important to identify which factor (e.g. p65, p53 and other regulators) is more dominant in a specific type of cancer. In fact, p53 was usually inactive due to gene mutation [45], while p65 was activated in response to the inflammatory tumor microenvironment [46]. Both the genetic and environment factors would regulate LPTS-dependent tumor progression.

## Conclusions

Taken together, we identified LPTS as a novel target of the transcription factor NF-κB. Modulation of NF-κB-LPTS axis might represent a promising target in cancer therapy.

## Abbreviations

CCK8: Cell counting kit-8
ChIP: Chromatin immunoprecipitation
EMSA: Electrophoretic mobility shift assay
FBS: Fetal bovine serum
HPV16: Human papillomavirus type 16
hTERT: human telomerase reverse transcriptase
IL-10: Interleukin-10
IL-1β: Interleukin-1β
IL-6: Interleukin-6
JNK: c-Jun NH2-terminal protein kinase
NF-κB: Nuclear factor-kappaB
PINX1: PIN2/TERF1-interacting Telomerase Inhibitor 1
qPCR: quantitative polymerase chain reaction
TNFα: Tumor necrosis factor α
